# Circulating tumor cells detection in tumor draining vein of breast cancer patients

**DOI:** 10.1038/s41598-019-54839-y

**Published:** 2019-12-03

**Authors:** Masaya Hattori, Hayao Nakanishi, Mayumi Yoshimura, Madoka Iwase, Akiyo Yoshimura, Yayoi Adachi, Naomi Gondo, Haruru Kotani, Masataka Sawaki, Nao Fujita, Yasushi Yatabe, Hiroji Iwata

**Affiliations:** 1 Department of Breast Oncology, Aichi Cancer Center, 1-1, Kanokoden, Chikusa, Nagoya 464-8681 Japan; 2 Department of Pathology and Molecular Diagnostics, Aichi Cancer Center, 1-1, Kanokoden, Chikusa, Nagoya 464-8681 Japan; 3 Laboratory of Pathology and Clinical Research, Aichi Cancer Center Aichi Hospital, 18 Kuriyada Kakemachi, Okazaki, 444-0011 Japan

**Keywords:** Breast cancer, Prognostic markers

## Abstract

Circulating tumor cells (CTCs) in tumor draining vein blood (DB) are potential sources for liquid biopsy. However, the identification of CTCs in DB of breast cancer has not been attempted. In this study, we investigated the feasibility of CTC detection in DB of breast cancer patients using a newly developed filtration-based microfluidic CTC detection device. Samples of peripheral vein blood (PB) and DB drawn from the lateral thoracic vein of the resected breast tissue were collected during the perioperative period. We investigated 41 breast cancer patients who underwent breast surgery with axillary lymph node dissection. DB was successfully collected in 36 patients (87.8%), with a mean amount of 0.85 ml. CTCs were detected in 58.3% of PB samples and 80.6% of DB samples. DB had significant higher number of CTCs compared with PB (*p* < 0.001). CTCs were detected in 75.0% of DB samples and 50.0% of PB samples from patients achieving pathological complete response after neoadjuvant chemotherapy. These results suggest that abundant CTCs are released into the DB of breast cancer patients, indicating that CTCs in DB would be alternative sources for liquid biopsy and potential indicators for monitoring of treatment response and prognosis in breast cancer patients.

## Introduction

Liquid biopsy for circulating tumor cells (CTCs) and cell-free circulating tumor DNA is a relatively new minimally invasive diagnostic method that has received much attention over the past decade^1,2^. CTCs have demonstrated utility for predicting metastasis and prognosis as well as for monitoring therapeutic effects in many cancers including colorectal, lung, prostate and breast cancers^3–6^. Furthermore, CTCs offer the opportunity to obtain information about a biological function in metastatic development^7,8^. Many studies have shown the clinical utility of CTCs in breast cancer^3,9–13^. The number of CTCs was reportedly as an independent prognostic factor^3,12^. In addition, expression of human epidermal growth factor receptor 2 (HER2) and other proteins in CTCs was reported to correlate with prognosis in metastatic breast cancer patients^14,15^. Despite the increased number of clinical studies of CTCs in breast cancer, CTC analysis remains challenging and has not been implemented in the clinical setting. One reason for the limited role of CTCs in clinical practice is the rarity of their release into peripheral vein blood (PB), especially in patients with early stage^11,16^. The current development of novel techniques and approaches to efficiently detect CTCs is expected to expedite their implementation in the clinical setting^16,17^. Notably, intraoperative CTC isolation from tumor draining vein blood (DB) is an exceptional opportunity to detect more abundant CTCs, as it allows easy access to draining veins in proximity to tumor flow in patients with colorectal cancer^18,19^ and lung cancer^20,21^. This strategy may help overcome the rarity problem of CTCs for liquid biopsy, despite the invasive nature of intraoperative blood collection. To date, however, no reports have examined CTC isolation from the draining vein in patients with breast cancer. One reason may be that the draining vein in breast cancer is too small in diameter to collect a sufficient volume of blood for successful detection of CTCs by standard detection methods.

Neoadjuvant chemotherapy (NAC) is currently a standard treatment option for primary breast cancer patients. NAC can aid in reducing tumor size before surgical treatment, as well as monitoring primary tumor response to chemotherapy and eradicating micrometastases, allowing some patients achieving pathological complete response (pCR), significantly increasing progression-free survival and overall survival^22^. pCR can be achieved in approximately 20–50% of breast cancer patients pretreated with NAC. However, approximately 20–30% of patients achieving pCR after NAC develop metastatic relapse. Recently, several investigators reported that CTCs could be detected even in patients achieving pCR after NAC^10,13,23,24^. Currently, the clinical significance of CTCs detected in pCR patients is unclear, and it is also unclear whether the CTCs are derived from primary tumors, harbored metastases or minimal residual disease in the whole body.

To address these issues, we conducted a pilot study to evaluate the feasibility and utility of CTC analysis in DB of breast cancer patients with or without neoadjuvant chemotherapy, using our recently developed filtration-based microfluidic CTC detection device^25^. The device contains a 3- dimensional (3D) metal filter that can trap CTCs based on size, after which we can cytologically detect CTCs using glass slide specimens stained by Papanicolau (Pap), immunocytochemistry (ICC), and immunofluorescence (IF) under light microscopy^25–27^. This system can isolate CTCs even in a small volume of blood sample and allow cytological evaluation of CTCs in permanent specimens that are difficult to observe by immunofluorescence microscopy under dark field conditions^26^. In the present study, we examined CTCs in PB collected one day before or just before surgery and in DB drawn from the lateral thoracic vein (LTV) of the resected breast tissues immediately after resection. We report that abundant CTCs were detected from DB of breast cancer patients compared with PB, and discuss the potential usefulness of CTCs in DB as a liquid biopsy assay in patients with breast cancer.

## Material and Methods

### Reagents

A mouse monoclonal antibody to human wide-spectrum (pan) cytokeratin (Clone, Oscar) was purchased from BioLegend (Dedham, MA, USA). Mouse monoclonal antibodies to human CD45 and estrogen receptor (ER) were obtained from DAKO (Carpinteria, CA, USA). Mouse monoclonal antibodies to human HER2 and human progesterone receptor (PR) were purchased from Abcam (Cambridge, UK) and Thermo Fisher Scientific (Waltham, MA, USA), respectively. For direct labeling of antibody, a Zenon Alexa fluor-488 (−568) mouse labeling kit was obtained from Invitrogen (Molecular Probe, Eugene, OR). Hoechst 33342 (Molecular Probes) was used for nuclear counterstaining. MCF-7 and BT-474 human breast cancer cells were used for cell-spiking experiments, and GLM-1 human gastric cancer cells, with high HER2 gene amplification, were used for demonstration of HER2 fluorescence *in situ* hybridization (FISH). MCF-7 and BT-474 cell lines were obtained from ATCC cell bank (Manassas, VA, USA) and GLM-1 cell line was established in our laboratory^28^. These cell lines were cultured in mediums as described previously^26^.

### Patients and blood sample collections

**Figure 1:**
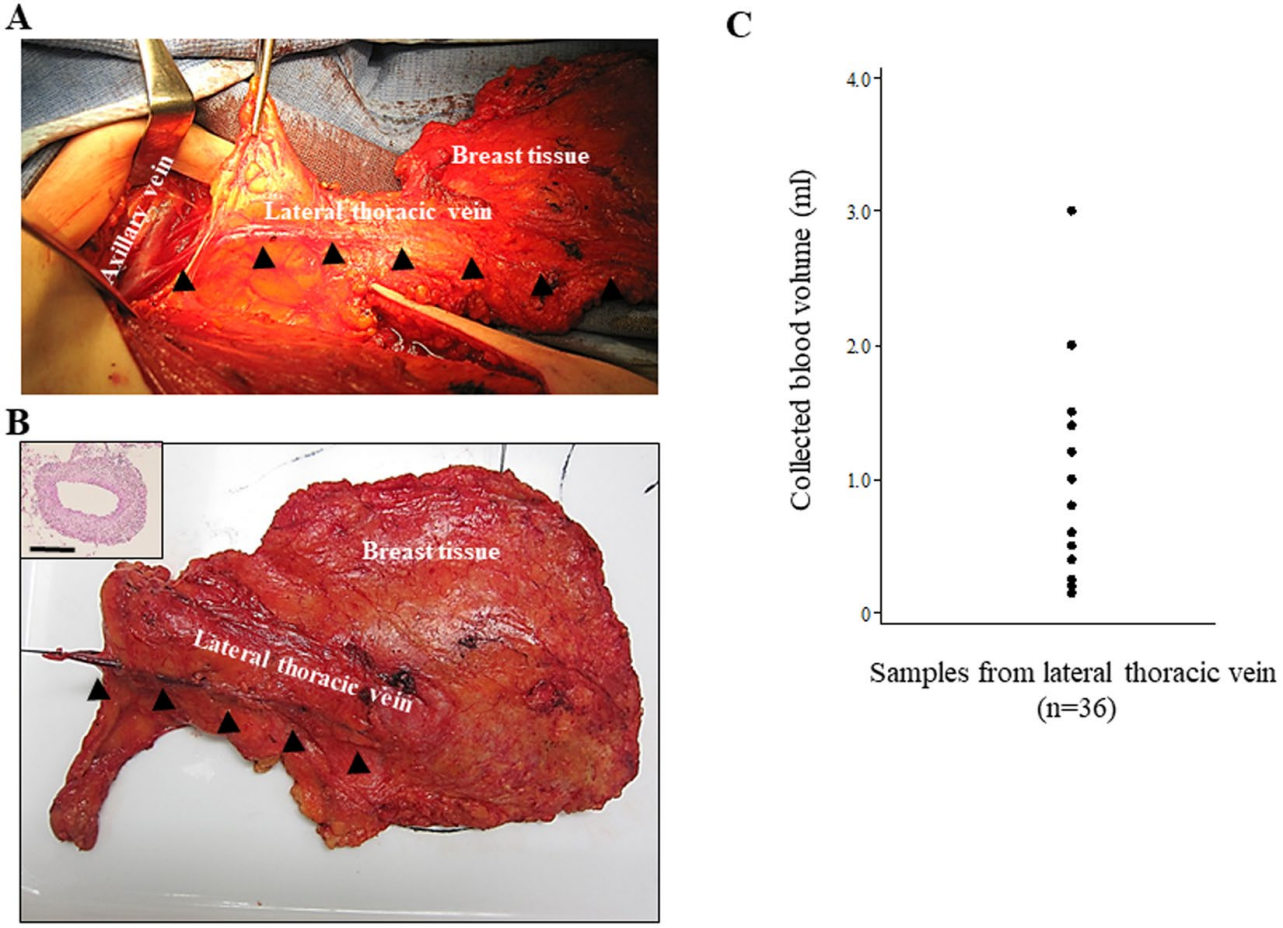
Collection of draining vein blood (DB) from breast cancer patients. (**A**) Blood from breast tumor tissue into the axillary vein via the lateral thoracic vein (LTV). Photomicrograph during operation. (**B**) Sampling of DB from LTV in the breast cancer patient immediately after resection. Inset shows histology (HE) of a LTV 1–2 mm in diameter. Bar = 1 mm. Arrowheads indicate the LTV. (**C**) Variation of volumes of collected DB samples in this study (n = 36).

Patients who underwent mastectomy or breast-conserving surgery plus axillary lymph node dissection (ALND) for non-metastatic breast cancer at Aichi Cancer Center were eligible for inclusion in this prospective pilot study. Patients who underwent breast surgery after NAC were included in this study. DB samples (≥0.2 ml) were collected into ethylenediaminetetraacetic acid (EDTA)-2Na tubes from the LTV of the resected breast tissue using a 26-gauge needle within a few minutes after resection (Fig. 1A,B) and were kept at room temperature and used for examination within 2 hours. PB samples (10 ml) were collected from a cubital vein one day before or just before surgery. PB samples from healthy volunteers (n = 20) were used as a negative control. The categories such as histological grade were determined from pathological findings based on core-needle biopsy samples or surgically resected specimens. ER and PR positivity were defined as score 2> (Allred score). Human epidermal growth factor-2 (HER2) positivity was defined as 3+ by immunohistochemistry or amplification of HER2 by fluorescent *in situ* hybridization (FISH). pCR was defined by the complete absence of invasive tumor cells in both breast and nodes under standard pathological examination. All procedures performed in the study were in accordance with the ethical standards of the institutional research committee and with the 1964 Helsinki declaration and its later amendments or comparable ethical standards. The study was approved by the institutional review board of Aichi Cancer Center (No. 2010-8-26 and No. 2016-1-157). Written informed consent was obtained from all individual participants included in the study.

### CTC enrichment and transfer to a glass slide from the filtration-based microfluidic device

**Figure 2:**
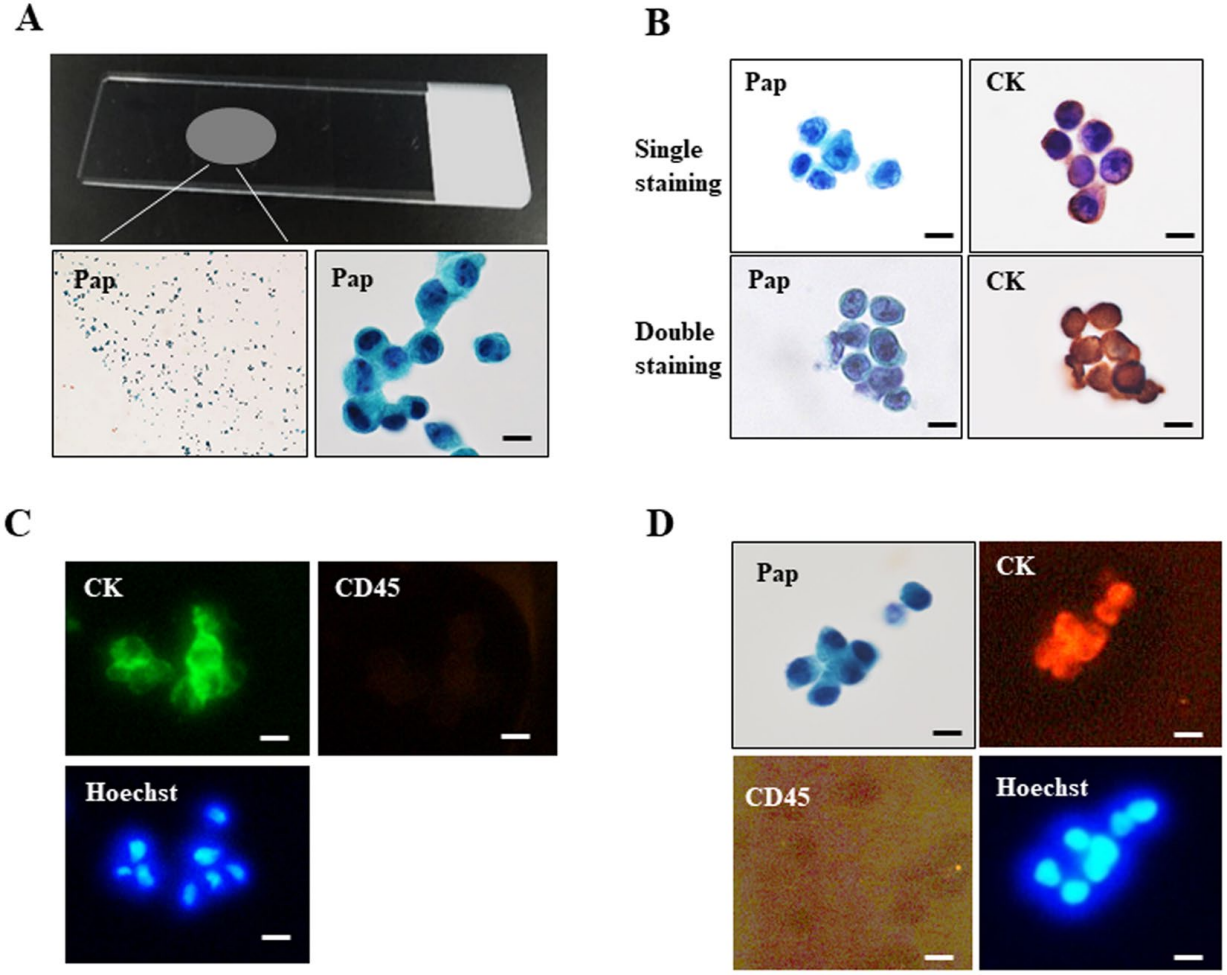
Cytology-based CTC detection methods using a glass slide. (**A**) CTC glass slide were prepared by transferring of tumor cells from the filter (top panel). Photomicrograph of resultant CTC glass slide stained by Pap (lower panels, left: low magnification view). (**B**) Pap staining and pan-cytokeratin immunocytochemistry (ICC) of MCF-7 cells on separate glass slides (top row). Double staining of Pap and pan-cytokeratin ICC of MCF-7 cells on the same glass slide (bottom row). (**C**) Conventional triple immunofluorescence (IF) (pan-cytokeratin/CD45/Hoechst) of MCF-7 cells stained directly on the filter. (**D**) Combination staining of Pap and triple IF of MCF-7 cells on the same glass slide. Bar = 10 µm.

A filtration-based microfluidic device with a 3D metal filter for rare cell enrichment (Optnics Precision Co., Ltd. Tochigi, Japan), as previously reported^27^, was utilized for CTC isolation in this study. The CTC detection procedure consists of three steps: (1) enrichment of CTCs by a filtration-based device containing a 3D metal filter^25^, (2) transfer of CTCs from the 3D metal filter to a glass slide (CTC glass slide) by brief centrifugation, and (3) subsequent cytological examination of the CTC glass slide by Pap staining and ICC. The methods for CTC enrichment and detection are largely the same as described previously^26,27^. Briefly, patient’s whole blood for PB (10 ml) and DB (≥0.2 ml) are diluted 10-fold with phosphate-buffered saline (PBS) and 0.5 mM EDTA (PBS/EDTA) and then filtered with the microfluidic device at a flow rate of 2.0–5.0 ml/min. After filtration, cells on the filter were fixed with 10% buffered formalin for 15–30 min, followed by washing with PBS/EDTA, and the 3D metal filter was then detached from the filtration-based device. The filter was then placed upside down onto a coated glass slide (MAS coat, Matsunami, Osaka, Japan) and the CTCs were quickly transferred to the glass slide by brief centrifugation (x2000 rpm, 20 sec) using a swing rotor (T5S32) at room temperature (Hitachi Himac CF16RX, Tokyo, Japan) or other mechanical pressure method. The resultant CTC glass slides were immediately fixed in 95% ethanol (≥60 min) for Pap staining, or in 95% ethanol followed by 10% buffered formalin (for 20 min) for ICC (Fig. 2A).

### CTC measurement

Blood samples were divided into two tubes and used to prepare two CTC slide specimens: one specimen was used for pan-cytokeratin ICC with hematoxylin nuclear counterstaining, and the other specimen was used for Pap staining to morphologically assist with the identification correct of CTC (Fig. 2B, top row). Alternatively, double staining was also available: the CTC slide was first stained by Pap and then de-stained by an ethanol series, followed by ICC for pan-cytokeratin on the same slide (Fig. 2B, bottom row). In either detection method, pan-cytokeratin positive cells with atypical morphology, such as high nuclear cytoplasmic ratio and prominent nucleoli revealed by Pap staining, were identified as CTCs. These diagnostic criteria for CTC based on cytology (cytokeratin ICC and Pap staining) can successful exclude leukocytes without using ICC for CD45.

### FISH analysis

Amplification of the c-erbB-2 gene was determined by a dual-color FISH method using a Passvision HER-2 DNA probe kit (Vysis Inc., Downers Grove, IL, USA) in accordance with the manufacturer’s protocol. The HER-2/neu-Spectrum Orange probe contains a DNA sequence specific for the c-erbB-2 gene locus on human chromosome 17. The chromosome 17 centromere (CEP17) green probe that hybridizes to the centromere region of chromosome 17 was used as a control. The nucleus was counterstained with 4′, 6-diamidino-2-phenylindole (DAPI). The slides were observed under a BX60 fluorescence microscope equipped with a digital camera (DP50, Olympus, Tokyo, Japan). A cell was considered to show amplification when the ratio of HER2 signals to CEP17 signals was ≥4.

### Statistical analysis

All statistical analyses were performed using STATA ver.12 (StataCorp, College Station, TX, USA). The significance of differences between CTCs in DB and PB were determined by applying the Student’s t-tests and the Wilcoxon signed rank test. Pearson correlation coefficients were used to evaluate whether CTC number in DB was correlated with CTC number in PB. Significance is determined if p-value < 0.05.

## Results

### Patient characteristics

Between January 2016 and May 2018, a total of 41 patients were enrolled in this pilot study. Of the 41 patients, 5 patients (12.2%) were excluded due to the small volume of DB (less than 0.2 ml with some blood clots). Unsuccessful blood collection was mainly due to the difficulty of correctly puncturing a small LTV (1–2 mm in diameter). The volume of collected DB ranged from 0.2 ml to 3.0 ml (median: 0.6 ml, mean: 0.85 ml) (Fig. 1C). In total, 36 patients with sufficient DB samples and PB samples for CTC evaluation were finally included in this pilot study. The clinical characteristics of these patients are summarized in Table 1. All patients were female, with a median age of 53 years old (range: 35–81). Of the 36 patients, 13 received surgery initially and 23 received surgery after NAC. All patients had node-positive disease, which was confirmed by needle aspiration cytology/biopsy before surgery or initiation of NAC. Most patients received neoadjuvant/adjuvant chemotherapy and all HER2-positive patients received anti-HER2 regimens. Of the 23 patients receiving NAC, 8 (34.7%) had achieved pCR.

**Table 1:** Clinical characteristics of the breast cancer patients included in this study.

	All patients, n (%)	Primary surgery patients, n (%)	NAC patients, n (%)
Total patients	36	13	23
Age, median (range), years	53 (35–81)	60 (44–81)	51 (35–73)
**Tumor stage**			
T0	1 (3)	0 (0)	1 (4)
T1	10 (28)	7 (54)	3 (13)
T2	19 (53)	6 (46)	13 (53)
T3	4 (11)	0 (0)	4 (17)
T4	1 (3)	0 (0)	1 (4)
**Lymph node status**			
1–3 lymph nodes	24 (67)	8 (62)	16 (70)
>3 lymph nodes	12 (33)	5 (38)	7 (30)
**Estrogen receptor**			
Positive	25 (69)	11 (85)	14 (61)
Negative	11 (31)	2 (15)	9 (39)
**Progesterone receptor**			
Positive	19 (53)	9 (69)	10 (43)
Negative	17 (47)	4 (31)	13 (57)
**HER2**			
Positive	10 (28)	1 (8)	9 (39)
Negative	26 (72)	12 (92)	14 (61)
**Histologic tumor grade**			
Grade 1	3 (8)	2 (15)	1 (4)
Grade 2	18 (50)	9 (69)	9 (39)
Grade 3	14 (39)	2 (15)	12 (52)
NA	1 (3)	0 (0)	1 (4)
**Neoadjuvant/adjuvant chemotherapy regimen**			
AC-T	21 (58)	7 (54)	14 (61)
AC-T + H	7 (19)	0 (0)	7 (30)
Others	5 (14)	3 (23)	2 (9)
None	3 (8)	3 (23)	0 (0)
**Pathologic complete response**			
Yes	—	—	8 (35)
No	—	—	15 (65)

Abbreviations: NAC, neoadjuvant chemotherapy; NA, not assessed; HER2, human epidermal growth factor-2; AC, doxorubicin and cyclophosphamide; T, taxane; H, Herceptin.

### Detection of CTCs

Tumor cells enriched by the filtration-based device were transferred from the 3D metal filter to a glass slide (CTC glass slide) and then subjected to subsequent cytological examination by Pap staining (Fig. 2A) and cytokeratin immunocytochemistry, using either single staining of two CTC glass slides or double staining of one CTC glass slide (Fig. 2B). Pap staining clearly showed malignant features of tumor cells, such as prominent nucleoli and a high nuclear/cytoplasmic ratio, whereas conventional triple IF staining of CTCs on the filter did not provide any morphological information under the dark field (Fig. 2C). This current staining method of using a CTC glass slide allowed for Pap staining with triple IF staining on the same slide, which can provide both morphological information and protein expression profiles (Fig. 2D).

### Subtype identification of CTCs by combination Pap (or ICC) with IF

**Figure 3:**
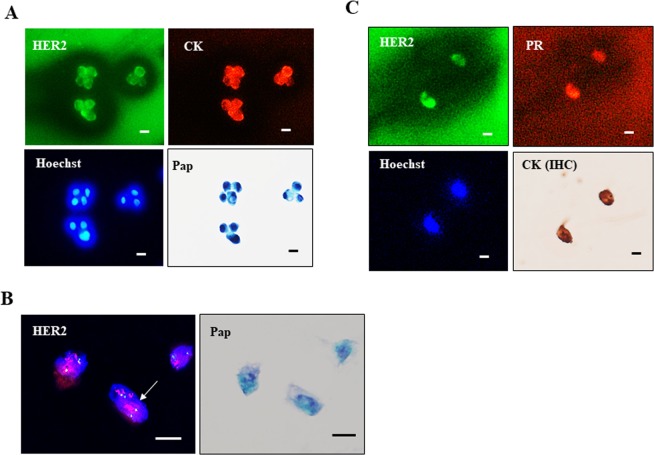
Subtyping of CTCs by the current cytology-based CTC detection method. (**A**) Triple IF (HER2/Pan-cytokeratin/Hoechst) of BT-474 cells, followed by Pap staining. (**B**) HER2 FISH of GLM-1 cells, followed by Pap staining. (**C**) Subtyping of CTCs in DB from a breast cancer patient by IF (HER2+/PR+/Hoechst), followed by pan-cytokeratin ICC. Bar = 10 µm.

The subtype (HER2/ER/PR) of breast cancer cells can be examined by combination staining of Pap (or ICC) with IF (HER2/ER/PR). In HER2-positive BT-474 breast cancer cells, for example, the expression of HER2/cytokeratin/Hoechst was examined first by triple IF followed by Pap staining (or Pap → IF), and the results confirmed the HER2 positivity of cytokeratin-positive BT-474 cells (Fig. 3A). The combination of HER2 FISH and Pap staining showed cluster type HER2 gene amplification of Pap-stained GLM-1 tumor cells (Fig. 3B). In a breast cancer patient with HER2+/ER+/PR+ subtype, simultaneous detection of HER2/PR expression by triple IF (HER2/PR/Hoechst) and cytokeratin expression of CTCs by ICC could be confirmed (Fig. 3C), indicating the potential utility of this method for the identification of breast cancer subtypes in the clinical setting.

### CTCs from DB and PB samples in breast cancer patients

**Figure 4:**
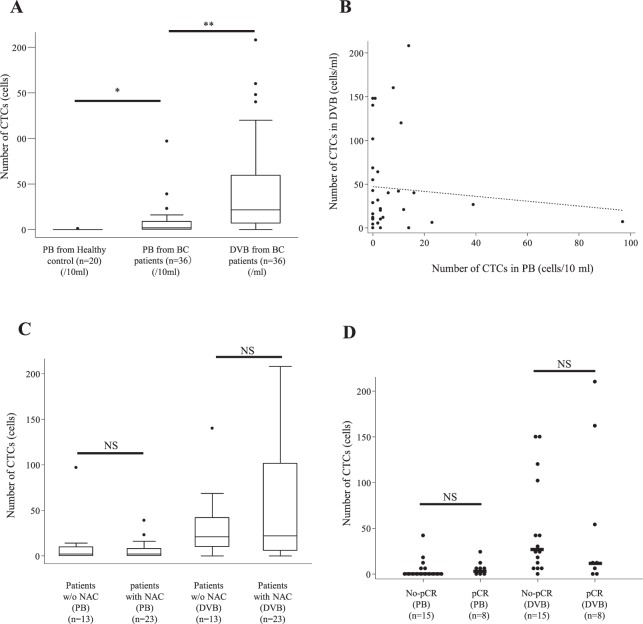
Number of CTCs measured in various blood samples from breast cancer (BC) patients. (**A**) Quantitative analysis of the number of CTCs in DB and peripheral blood (PB) in BC patients and PB in healthy volunteers. *p = 0.03 (PB in patients vs. PB in healthy volunteers), **p < 0.01 (DB vs. PB in BC patients). (**B**) Correlation of CTC number between PB and DB in BC patients. No correlation was observed (correlation coefficient: r = −0.0875). (**C**) Comparison of CTC numbers between PB and DB from BC patients with and without (w/o) neoadjuvant chemotherapy (NAC). No significant difference was observed between BC patients with and without NAC both in both PB (per 10 ml) and DB (per ml). (**D**) Comparison of CTC numbers in PB (per 10 ml) and DB (per ml) between BC patients with residual disease to NAC (No-pCR) and pathological complete response (pCR). No significant differences in the numbers of CTCs detected in PB and DB between No-PCR and pCR. *p = 0.39 (No-pCR PB vs. pCR PB), **p = 0.85 (No-pCR DB vs. pCR DB).

CTCs were detected in PB samples in 21 patients (58.3%), and the median and mean numbers of CTCs in PB samples were 2.0 and 7.3 (per 10 ml, range: 0–97), respectively. We detected a significantly higher rate and number of CTCs in PB samples from breast cancer patients compared with samples from 20 healthy controls (*p* < 0.01 and *p* = 0.03, respectively) (Fig. 4A). In contrast, CTCs were detected in DB samples in 31 patients (80.6%). As the mean volume of DB samples was 0.85 ml, CTC numbers in DB samples were extrapolated to values for 1.0 ml. The median and mean numbers of CTCs identified in DB samples were 21.5 and 45.2, respectively (per ml, range: 0–208). The detection rate and number of CTCs were significantly higher in DB samples than in PB samples (*p* < 0.01 and *p* < 0.001, respectively) (Fig. 4A). Among the 15 patients in which CTCs were not detected in PB samples, we detected CTCs in DB samples from 12 of these patients. However, there was no correlation in the distribution of CTC numbers between DB samples (per ml) and PB samples (per 10 ml) (r = −0.0875) (Fig. 4B). CTCs were detected from DB samples in 11 patients (84.6%) who received initial surgery and in 20 patients (86.9%) who received NAC. The median numbers of CTCs identified from DB samples were 21.0 and 22.0 (per ml), respectively. These results indicate that there was no significant difference in the detection of CTCs from DB between initial surgery patients and NAC-treated patients (*p* = 0.85) (Fig. 4C).

### CTCs from DB and PB samples in NAC patients

**Figure 5:**
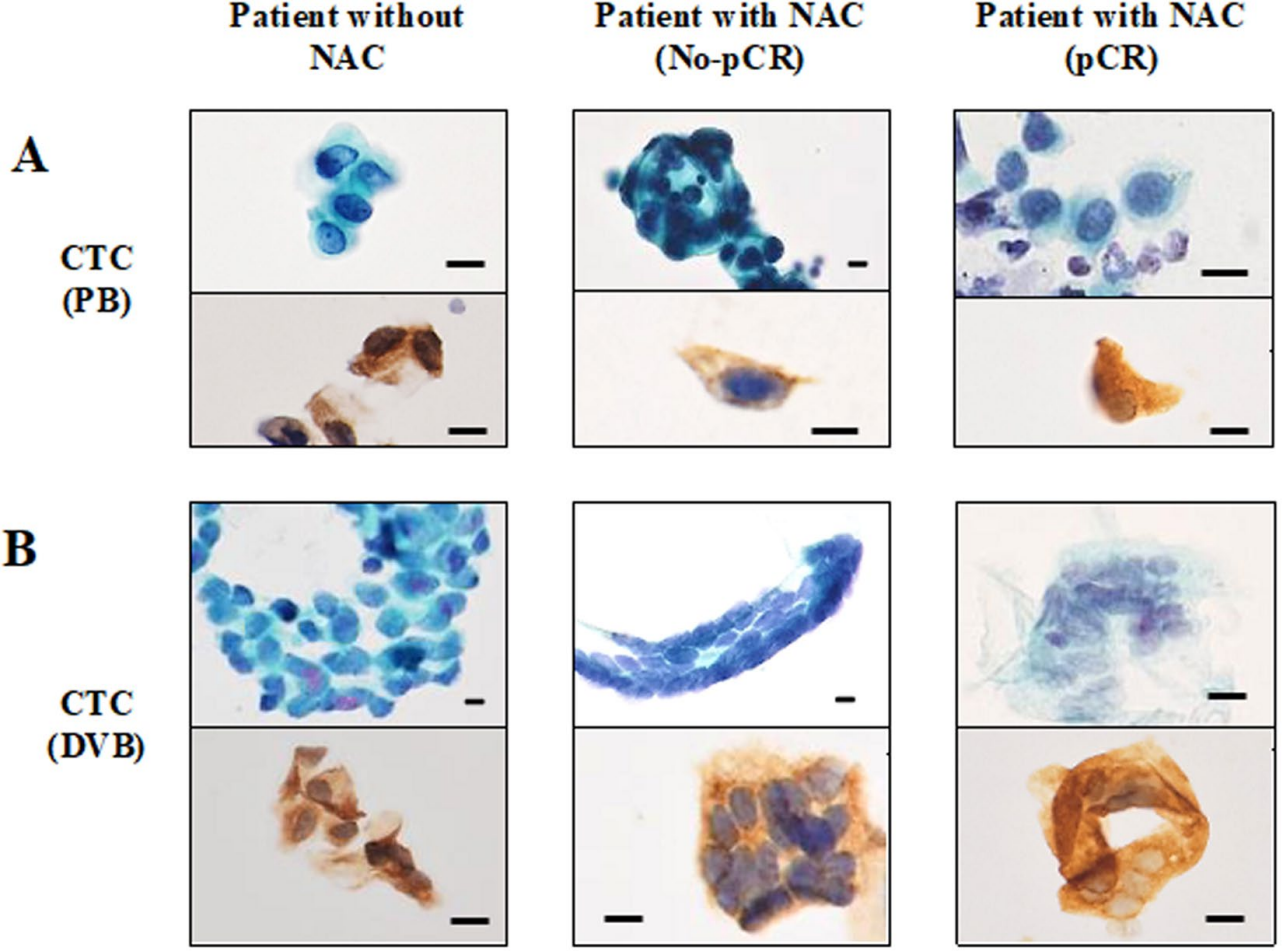
Morphology of CTCs in PB and DB from the same BC patients stained by Pap and cytokeratin ICC. (**A**) Representative CTCs in PB from BC patients without NAC, with No-pCR and with pCR after NAC. (**B**) Representative CTCs in DB from BC patients without NAC, with No-pCR and with pCR after NAC. CTCs obtained from patients with pCR after NAC exhibited some degenerative changes such as swollen nuclei and rough nuclear chromatin. Bar = 10 µm.

In patients treated with NAC, CTCs were detected from both DB and PB samples in patients with residual disease (No-pCR) as well as in patients that achieved pCR. Of the 15 NAC patients with residual disease, CTCs were detected in DB samples from 14 patients (93.3%) and in PB samples from 8 patients (53.3%), with a median of identified CTCs of 16.0 (per ml) and 1.0 (per 10 ml), respectively. Of the 8 NAC-treated patients who achieved pCR, CTCs were detected from DB samples in 6 patients (75.0%) and from PB samples in 4 patients (50.0%), with a median number of identified CTCs of 11.0 (per ml) and 3.0 (per 10 ml), respectively (Supplementary Table 1). There was no significant difference in the numbers of CTCs identified both from DB and PB samples between patients with residual disease and those achieving pCR (*p* = 0.85 and *p* = 0.39, respectively) (Fig. 4D). CTCs in DB and PB samples of patients achieving pCR appeared to be viable, but showed some degenerative changes in some cases based on the morphology revealed by Pap staining (Fig. 5A,B).

## Discussion

Here we used a recently developed filtration-based microfluidic CTC detection device to successfully detect significantly higher numbers of CTCs in DB samples obtained from the LTV of the breast cancer patients than in PB samples. Previous studies have examined CTCs in DB from colon and lung cancer patients^18,21^, and the presence of tumor cells in DB has been reported in experimental rat mammary tumor models^29^. To the best of our knowledge, this is the first report of the detection and characterization of CTCs in DB from breast cancer patients. Since collection of DB from the LTV was taken from the resected breast tissue, this method is non-invasive compared with the low invasive nature of typical liquid biopsies. Because the blood samples were drawn from LTV after resection, the volume of aspirated DB was small, ranging from 0.2 ml to 3.0 ml in our study, but the number of identified CTCs from DB was much higher (median: 21.5 per ml) compared with CTCs identified from PB (median: 2.0 per 10 ml). These obtained greater numbers of CTCs are potentially sufficient for downstream analysis^1,8,21^. Furthermore, contamination with endothelial cells due to the injury of small vessels by the needle was negligible, and the morphology of CTCs generally indicated healthy cells as evaluated by Pap staining^30^. These findings demonstrate the feasibility and usefulness of DB as a potential source for CTCs for liquid biopsy.

Compared with the majority of CTC detection methods, our current CTC detection device with CTC-attached glass slides has the following unique characteristics: (1) Unlike most immunofluorescence staining criteria, such as cytokeratin+/CD45−/DAPI+ judged under dark field as reported previously^1,3^, in the current study, CTC can be detected by the combination of Pap staining and ICC for pan-cytokeratin on a permanent glass slide under light microscopy without CD45 staining^26^. (2) An advantage of this cytology-based method is the acquisition of detailed cytological information for CTCs with atypical morphology assessed by Pap staining, allowing detection of CTCs with epithelial-mesenchymal transition and CTCs damaged by chemotherapy, based on morphological features such as enlarged nuclei/prominent nucleoli, apoptosis and M phase-arrest, as reported previously^26^. (3) The use of a CTC glass slide makes possible further combination staining of Pap (or cytokeratin ICC) and triple IF, such as HER2/ER(PR)/Hoechst 33342 staining or HER2 FISH, which allows subtype identification of CTCs on the same slide^27^.

Another interesting finding in this study is the detection of CTCs in DB samples not only in patients with residual disease (No-pCR), but also in patients achieving pCR after NAC, although the CTC numbers tended to be lower in patients with pCR compared with patients without NAC and patients with no-pCR. Several investigators previously reported the detection of CTCs in PB samples in breast cancer patients achieving pCR after NAC^13^, but no study has yet reported identification of CTCs in DB. Our study demonstrated the presence of a significant number of CTCs in DB in patients achieving pCR at higher incidence. It has reported that many tumor cells which detach from the primary site enter into the bloodstream in rat mammary tumor models and are detectable in tumor venous blood^29^. CTCs in PB would be influenced by reservoirs harboring metastasis in addition to the primary site, whereas CTCs in DB presumably reflect the cells directly derived from the primary tumor^1,19,21^. In this respect, it is worth noting that the numbers of CTCs in DB are increased through surgical intervention-driven mobilization of tumor cells in the primary tumor^31–33^. Therefore, it is likely that CTCs in DB better reflect the primary tumor state. The presence of CTCs in DB of patients achieving pCR might suggest that pCR is not “true pCR”, but “limited pCR”, with some false-negative risk under standard pathological examination. We further demonstrated that the morphology of CTCs in DB in patients with pCR appeared viable like CTCs in patients with residual disease, despite some degenerative changes detected by Pap staining in CTCs of pCR cases. Recently, Kasimir *et al*. reported that CTCs after NAC showed stem cell-like features, suggesting the possibility that the remaining CTCs after NAC are resistant to chemotherapy^34^. These findings suggest that CTCs in DB are more reliable and useful indicators for the primary tumor’s sensitivity and resistance to NAC and could have both prognostic and predictive significance. However, we did not observe a difference in clinical outcome between pCR patients with and without CTCs detected in DB. This is probably due to the short follow-up period of this study (less than 1 year). We are now continuing a longer follow-up for this study to clarify these points.

Major limitation of this pilot study is that DB (LTV)-based CTC method only could be performed for only one time at operation, which lost the features of multiple times of liquid biopsy. Theoretically, PB-based CTC could collect any cancer cells from all the primary and metastatic tumors in breast cancer patients, whereas DB-based CTC may collect cancer cells only from primary tumors. Another limitation of this study is the small blood volume (0.2–3.0 ml) collected from the draining vein of the patients. However, collection of more than 3 ml of DB from the LTV would be possible if the blood was drawn prior to the ligation of the LTV during operation. Furthermore, current study is a biased study in terms of the enrollment of only patients who received ALND. A further non-biased study including patients with and without ALND is possible and will be needed to confirm our present findings.

In conclusion, we demonstrated for the first time that DB from the LTV of breast cancer patients is a potential rich source of CTCs that better reflects the primary tumor state, with no risk, and can be sufficient for analysis of CTCs. Therefore, despite some limitations described above, the detection of CTCs in DB would be a potentially powerful tool to overcome the problem of insufficient liquid biopsy in clinical settings. Furthermore, even in patients achieving pCR after NAC, there still remain a significant number of CTCs in DB, which may lead to metastasis. These findings suggest that CTCs in DB could be a potential predictor for relapse, chemosensitivity and indicator for treatment decision in patients receiving NAC. Further large-scale prospective studies of CTCs in DB in breast cancer patients with NAC are needed.

## Supplementary information

Supplementary Table 1

## Data Availability

The datasets during and/or analysed during the current study are available from the corresponding author on reasonable request.

## References

[1] Bardelli A, Pantel K (2017). Liquid Biopsies, What We Do Not Know (Yet). Cancer cell.

[2] Jordan NV (2016). HER2 expression identifies dynamic functional states within circulating breast cancer cells. Nature.

[3] Cristofanilli M (2004). Circulating tumor cells, disease progression, and survival in metastatic breast cancer. The New England journal of medicine.

[4] Scher HI (2009). Circulating tumour cells as prognostic markers in progressive, castration-resistant prostate cancer: a reanalysis of IMMC38 trial data. The lancet oncology.

[5] Krebs MG (2011). Evaluation and prognostic significance of circulating tumor cells in patients with non-small-cell lung cancer. Journal of clinical oncology: official journal of the American Society of Clinical Oncology.

[6] Cohen SJ (2008). Relationship of circulating tumor cells to tumor response, progression-free survival, and overall survival in patients with metastatic colorectal cancer. Journal of clinical oncology: official journal of the American Society of Clinical Oncology.

[7] Pantel K, Alix-Panabieres C (2016). Functional Studies on Viable Circulating Tumor Cells. Clinical chemistry.

[8] Alix-Panabieres C, Pantel K (2017). Characterization of single circulating tumor cells. FEBS letters.

[9] Lucci A (2012). Circulating tumour cells in non-metastatic breast cancer: a prospective study. The lancet oncology.

[10] Riethdorf S (2017). Prognostic Impact of Circulating Tumor Cells for Breast Cancer Patients Treated in the Neoadjuvant “Geparquattro” Trial. Clinical cancer research: an official journal of the American Association for Cancer Research.

[11] Pierga JY (2008). Circulating tumor cell detection predicts early metastatic relapse after neoadjuvant chemotherapy in large operable and locally advanced breast cancer in a phase II randomized trial. Clinical cancer research: an official journal of the American Association for Cancer Research.

[12] Pierga JY (2012). High independent prognostic and predictive value of circulating tumor cells compared with serum tumor markers in a large prospective trial in first-line chemotherapy for metastatic breast cancer patients. Annals of oncology: official journal of the European Society for Medical Oncology/ESMO.

[13] Pierga JY (2017). Circulating tumour cells and pathological complete response: independent prognostic factors in inflammatory breast cancer in a pooled analysis of two multicentre phase II trials (BEVERLY-1 and -2) of neoadjuvant chemotherapy combined with bevacizumab. Annals of oncology: official journal of the European Society for Medical Oncology/ESMO.

[14] Mostert B (2015). Gene expression profiles in circulating tumor cells to predict prognosis in metastatic breast cancer patients. Annals of oncology: official journal of the European Society for Medical Oncology/ESMO.

[15] Peeters DJ (2014). Detection and prognostic significance of circulating tumour cells in patients with metastatic breast cancer according to immunohistochemical subtypes. British journal of cancer.

[16] Alix-Panabieres C, Pantel K (2014). Challenges in circulating tumour cell research. Nature reviews. Cancer.

[17] Kowalik A, Kowalewska M, Gozdz S (2017). Current approaches for avoiding the limitations of circulating tumor cells detection methods-implications for diagnosis and treatment of patients with solid tumors. Translational research: the journal of laboratory and clinical medicine.

[18] Rahbari NN (2012). Compartmental differences of circulating tumor cells in colorectal cancer. Annals of surgical oncology.

[19] Wind J (2009). Circulating tumour cells during laparoscopic and open surgery for primary colonic cancer in portal and peripheral blood. European journal of surgical oncology: the journal of the European Society of Surgical Oncology and the British Association of Surgical Oncology.

[20] Okumura Y (2009). Circulating tumor cells in pulmonary venous blood of primary lung cancer patients. The Annals of thoracic surgery.

[21] Murlidhar V (2017). Poor Prognosis Indicated by Venous Circulating Tumor Cell Clusters in Early-Stage Lung Cancers. Cancer research.

[22] Cortazar P (2014). Pathological complete response and long-term clinical benefit in breast cancer: the CTNeoBC pooled analysis. Lancet.

[23] Bidard FC (2018). Circulating Tumor Cells in Breast Cancer Patients Treated by Neoadjuvant Chemotherapy: A Meta-analysis. Journal of the National Cancer Institute.

[24] Hall C (2015). Circulating Tumor Cells After Neoadjuvant Chemotherapy in Stage I-III Triple-Negative Breast Cancer. Annals of surgical oncology.

[25] Yusa A (2014). Development of a new rapid isolation device for circulating tumor cells (CTCs) using 3D palladium filter and its application for genetic analysis. PloS one.

[26] Adachi Yayoi, Yoshimura Mayumi, Nishida Keiko, Usuki Hisanobu, Shibata Keiko, Hattori Masaya, Kondo Naoto, Yatabe Yasushi, Iwata Hiroji, Kikumori Toyone, Kodera Yasuhiro, Nakanishi Hayao (2017). Acute phase dynamics of circulating tumor cells after paclitaxel and doxorubicin chemotherapy in breast cancer mouse models. Breast Cancer Research and Treatment.

[27] Dejima H (2018). Detection of abundant megakaryocytes in pulmonary artery blood in lung cancer patients using a microfluidic platform. Lung Cancer.

[28] Yokoyama H (2006). Molecular basis for sensitivity and acquired resistance to gefitinib in HER2-overexpressing human gastric cancer cell lines derived from liver metastasis. British journal of cancer.

[29] Butler TP, Gullino PM (1975). Quantitation of cell shedding into efferent blood of mammary adenocarcinoma. Cancer research.

[30] Rahbari NN (2017). Prognostic value of circulating endothelial cells in metastatic colorectal cancer. Oncotarget.

[31] Weitz J (1998). Dissemination of tumor cells in patients undergoing surgery for colorectal cancer. Clinical cancer research: an official journal of the American Association for Cancer Research.

[32] Hashimoto M (2014). Significant increase in circulating tumour cells in pulmonary venous blood during surgical manipulation in patients with primary lung cancer. Interactive cardiovascular and thoracic surgery.

[33] Martin OA, Anderson RL, Narayan K, MacManus MP (2017). Does the mobilization of circulating tumour cells during cancer therapy cause metastasis? *Nature reviews*. Clinical oncology.

[34] Kasimir-Bauer S (2016). Does primary neoadjuvant systemic therapy eradicate minimal residual disease? Analysis of disseminated and circulating tumor cells before and after therapy. Breast cancer research: BCR.

