# Supplementary material for: Circulating tumor cells detection in tumor draining vein of breast cancer patients
Source: Sci Rep. 2019 Dec 3;9:18195. doi: 10.1038/s41598-019-54839-y (PMC6890763; doi:10.1038/s41598-019-54839-y)
Supplement: Supplementary file 1 — Supplementary Table 1 [file 41598_2019_54839_MOESM1_ESM.pdf]

Supplementary information

TITLE:

Circulating tumor cells detection in tumor draining vein of breast cancer patients

AUTHORS:

Masaya Hattori[1], Hayao Nakanishi[2, 3], Mayumi Yoshimura[2], Madoka Iwase[1], Akiyo Yoshimura[1], Yayoi Adachi[1], Naomi Gondo[1], Haruru Kotani[1], Masataka Sawaki[1], Nao Fujita[2], Yasushi Yatabe[2] and Hiroji Iwata[1]

AFFILIATIONS:

[1] Department of Breast Oncology, Aichi Cancer Center, 1-1 Kanokoden, Chikusa, Nagoya, 464-8681, Japan

[2] Department of Pathology and Molecular Diagnostics, Aichi Cancer Center, 1-1, Kanokoden, Chikusa, Nagoya 464-8681, Japan

[3] Laboratory of Pathology and Clinical Research, Aichi Cancer Center Aichi Hospital, 18 Kuriyada Kakemachi, Okazaki, 444-0011, Japan

CORRESPONDING AUTHOR:

Masaya Hattori, MD

1-1 Kanokoden, Chikusa, Nagoya, 464-8681, Japan

Phone: +81-52-762-6111

Fax: +81-52-764-2963

Email: mhattori@aichi-cc.jp

Supplementary Table 1.

CTC numbers and survival outcome in 8 patients who achieved pathological complete response

Patient No.	Age	ER and/or PR	HER2	DB volume (ml)	CTC numbers in DB	CTC numbers in PB (/10ml)	Survival outcome
1	52	Positive	Positive	2.0	0	0	No recurrence
2	57	Negative	Positive	1.0	11	0	No recurrence
3	36	Positive	Negative	0.2	11	0	No recurrence
4	44	Negative	Negative	0.2	0	3	No recurrence
5	64	Negative	Positive	0.6	6	3	No recurrence
6	58	Negative	Positive	0.2	32	8	No recurrence
7	48	Positive	Positive	0.3	52	14	No recurrence
8	67	Negative	Negative	3.0	19	23	No recurrence

Abbreviations: ER, estrogen receptor; PR, progesterone receptor; HER2, human epidermal growth factor-2; DB, draining vein blood; PB, peripheral blood, CTC circulating tumor cell.
